# Antimicrobial Stewardship Programme

**DOI:** 10.1093/jacamr/dlz011

**Published:** 2019-04-12

## Abstract

**Graphical Abstract:**
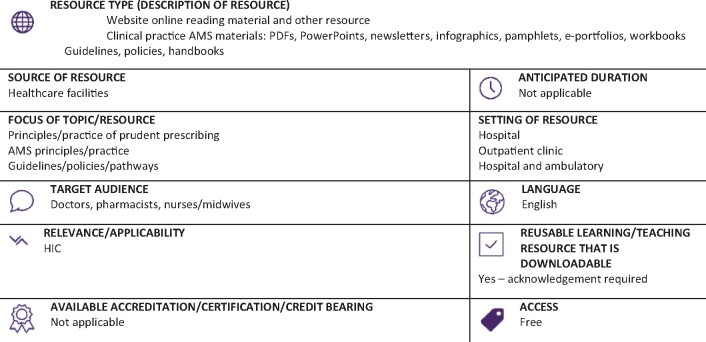

**Resource web link:** **https://www.nebraskamed.com/for-providers/asp** (Full classification scheme available at: http://bsac.org.uk/wp-content/uploads/2019/03/Educational-resource-review-classification-scheme.pdf)

**WHO region and country (World Bank):** North America, USA (HIC)

## Peer review commentary

Although this is quite an institution-specific resource based on a North American healthcare setup, there is a wealth of detail contained within it that will be of interest to those looking to develop guidelines; the antimicrobial guidebook is detailed and covers a range of clinical and laboratory considerations. There are some educational videos on outpatient prescribing that will be relevant in all countries; the guidelines and pathways are available in an App as well as the website.

The Pharmacokinetic Packet is aimed at pharmacists who provide advice on dosing and monitoring of antibiotics requiring therapeutic drug monitoring. Some of the information on the website appears to be a little dated now, with references up to 2011–12, but not later.

Nevertheless, the information and recommendations are based on robust evidence, are well written and will be a useful reference to those mainly based in HICs where there are laboratory and diagnostic services available to support an antimicrobial stewardship service set up like this one.

